# Two Pore Channel 2 Differentially Modulates Neural Differentiation of Mouse Embryonic Stem Cells

**DOI:** 10.1371/journal.pone.0066077

**Published:** 2013-06-12

**Authors:** Zhe-Hao Zhang, Ying-Ying Lu, Jianbo Yue

**Affiliations:** Department of Physiology, University of Hong Kong, Hong Kong, China; Baylor College of Medicine, United States of America

## Abstract

Nicotinic acid adenine dinucleotide phosphate (NAADP) is an endogenous Ca^2+^ mobilizing nucleotide presented in various species. NAADP mobilizes Ca^2+^ from acidic organelles through two pore channel 2 (TPC2) in many cell types and it has been previously shown that NAADP can potently induce neuronal differentiation in PC12 cells. Here we examined the role of TPC2 signaling in the neural differentiation of mouse embryonic stem (ES) cells. We found that the expression of TPC2 was markedly decreased during the initial ES cell entry into neural progenitors, and the levels of TPC2 gradually rebounded during the late stages of neurogenesis. Correspondingly, TPC2 knockdown accelerated mouse ES cell differentiation into neural progenitors but inhibited these neural progenitors from committing to neurons. Overexpression of TPC2, on the other hand, inhibited mouse ES cell from entering the early neural lineage. Interestingly, TPC2 knockdown had no effect on the differentiation of astrocytes and oligodendrocytes of mouse ES cells. Taken together, our data indicate that TPC2 signaling plays a temporal and differential role in modulating the neural lineage entry of mouse ES cells, in that TPC2 signaling inhibits ES cell entry to early neural progenitors, but is required for late neuronal differentiation.

## Introduction

The in vitro generation of neural cells from ES cells promises to produce an almost unlimited supply of cells suitable for cell-based replacement therapies in the nervous system [1]–[5]. The most widely used method to trigger neural differentiation is to induce embryoid body (EB) formation followed by retinoic acid (RA) treatment [5], [6], or, to culture ES cells with stroma conditioned medium [7], [8]. Several studies have been able to direct ES cell differentiation and to generate specific neuronal populations, including spinal cord motor neurons, dorsal interneurons, cerebellar Purkinje and granule cells, and midbrain dopaminergic neurons [9], [10]. Because ES cells are pluripotential and readily differentiate into almost any cell type in suspension culture, the efficiency of neural induction by these methods is low and the final cultures are always a heterogenous mixture of various cell types [1]. A simple and efficient way to induce ES cells into neural precursors and subsequently generate neuronal and glia cells is to culture ES cells in an adherent monolayer in defined medium [1], [2]. In this method, ES cells are cultured in defined serum-free and feeder-free conditions, in the absence of bone morphogenetic protein (BMP) and Wnts signals. In these conditions, ES cells undergo neural commitment through an autocrine fibroblast growth factor (FGF) signaling mechanism. This method results in a more efficient neural differentiation. Yet, around 40% of cells still resist neural specification and adopt nonneural fates [1], [2]. Therefore, to more efficiently induce neural commitment of ES cells, it is essential to define novel cellular and molecular events involved in neural differentiation.

Mobilization of intracellular Ca^2+^ stores is involved in almost all the aspects of cellular processes, e.g. neural differentiation [11]–[14]. Nicotinic adenine acid dinucleotide phosphate (NAADP) is one of the most potent endogenous Ca^2+^ mobilizing messengers. NAADP is formed by a base-exchange reaction that replaces the nicotinamidemoiety of NADP with nicotinic acid and is catalyzed by ADP-ribosyl cyclases (ARCs). Two enzymes have so far been shown to be capable of synthesizing NAADP from NADP in vitro, *Aplysia* ARC and CD38. Endogenous NAADP levels can be modulated by a variety of extracellular stimuli. NAADP mobilizes Ca^2+^ from an acidic lysosomes-related store, which can be subsequently amplified into global Ca^2+^ waves by CICR from ER/SR via IP_3_ receptors (IP_3_Rs) or ryanodine receptors (RyRs) [15], [16]. Recently, two pore channel 2 (TPC2), a new member of the superfamily of voltage-gated ion channels containing 12 putative transmembrane segments, has been demonstrated to be the NAADP receptors in many cell types. TPC2 is located on lysosomal membranes when expressed in several cell types. TPC2 overexpression promotes NAADP-induced Ca^2+^ release from lysosome-related stores, whereas ablating or knocking-down TPC2 expression blocked NAADP-induced Ca^2+^ release [17]–[24]. Yet it has been recently shown that NAADP does not directly bind to TPC2 [25]–[27]. In addition, TPC1, TRPML1, TRPM2, or RyRs has been reported to be NAADP receptor in different cell types [28]–[33].

NAADP/Ca^2+^ signaling pathway regulates diverse cellular processes, including fertilization [34], [35], receptor activation in lymphocytes [36], insulin secretion in pancreatic islets [37], hormonal signaling in pancreatic acinar cells [38], platelet activation [39], cardiac muscle contraction [40], and blood pressure control [41]. Since its discovery, NAADP has been found to regulate neuronal functions [42]. NAADP elicits Ca^2+^ changes in different neuronal preparations, including primary cultures of neuron, astrocytes, buccal ganglion of *Aplysia*, and frog neuromuscular junction [43]–[47]. NAADP has also been shown to induce neurite outgrowth [48], regulate neurotransmission [44], and modulate neuron polarization [49]. Moreover, it has been shown that NAADP, but not IP_3_ or cADPR, induces neuron differentiation of PC12 cells, although all three messengers can effectively activate Ca^2+^ signals [50]. Yet, the role and mechanism of NAADP/TPC2 signaling in embryonic neurogenesis remain to be determined. Here we examined the role of NAADP/TPC2 signaling in the neural differentiation of mouse ES cells and found that TPC2 signaling inhibits ES cell entry to early neural progenitors, but is required for late neuronal differentiation.

## Results

### Characterization of NAADP/TPC2/Ca^2+^ Signaling in Mouse ES Cells

**Figure 1:**
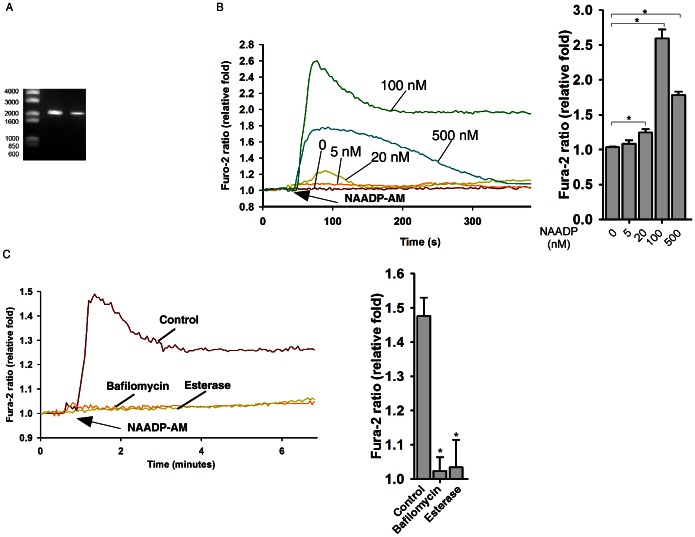
TPC2 signaling in mouse ES cells. (**A**) Expressions of TPC2 mRNAs in D3 ES cells were determined by RT-PCR. (**B**) NAADP-AM induced a Ca^2+^ increase in TPC2 overexpressing ES cells in a bell-shaped concentration response curve. (**C**) Inhibition of Ca^2+^ response triggered by NAADP-AM (50 nM) by bafilomycin A1 (100 nM) and esterase (50 units/ml). Data quantifications of [Ca^2+^]_i_ peak induced by drug treatment in (B) and (C) are expressed as mean ± S.E., n = 30–40 cells. The * symbols indicate the results of *t* Test analysis, *p*<0.05.

To assess a potential contribution of NAADP signaling to ES cell fate decisions, we first surveyed the expression of TPC2 in several types of ES cells by RT-PCR. As shown in Figure 1A, TPC2 mRNA was detected in D3 ES cells by RT-PCR using two different pairs of mouse TPC2 primers (Figure 1A). Similar results were found in 46C and R1 ES cells (Figure S1). Next, we examined whether NAADP-AM, a cell-permeable NAADP agonist, could trigger Ca^2+^ release in mouse ES cells. As shown in Figure 1B, NAADP-AM triggered Ca^2+^ release in D3 ES cells at concentrations ranging from 5 nM to 500 nM. The bell shaped concentration response curve well fits the model of NAADP-mediated Ca^2+^ responses in pancreatic cells, heart cells and T cells. Preincubation of NAADP-AM with esterase (50 units/ml) blocked NAADP-AM triggered Ca^2+^ release (Figure 1C), indicating that NAADP-AM, not NAADP itself, can penetrate the cell membrane to trigger Ca^2+^ release. In addition, NAADP-AM triggered Ca^2+^ releases in mouse ES cells were blocked by H^+^ ATPase inhibitor, bafilomycin A1 (100 nM) (Figure 1C). These data indicate that the mouse ES cells are responsive to NAADP and possess the key components of NAADP-signaling pathway.

**Figure 2:**
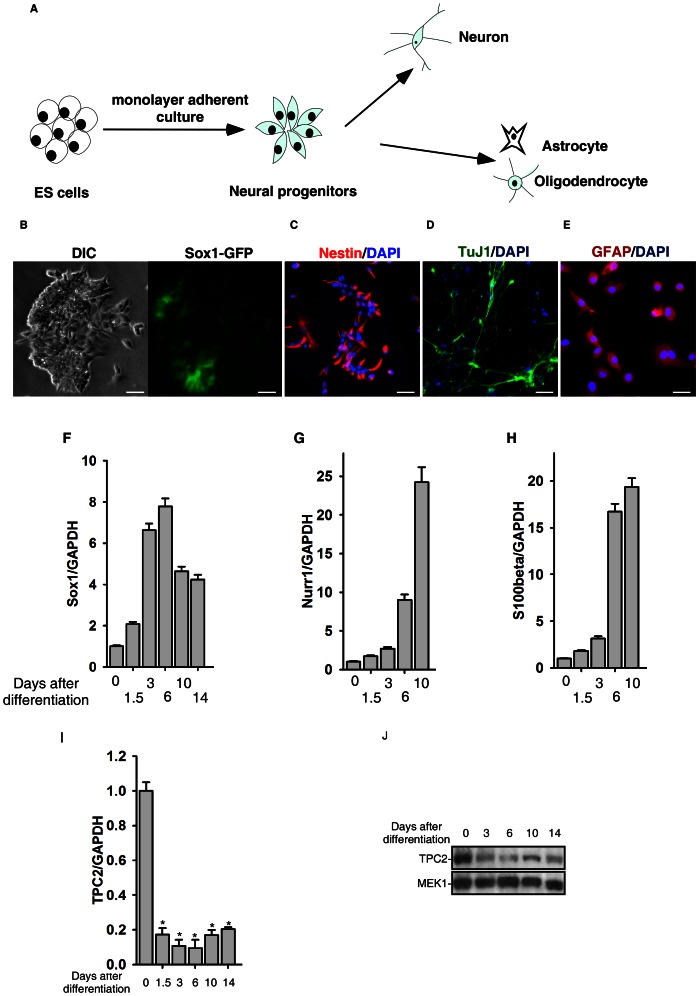
Expression pattern of TPC2 during in vitro neural differentiation of mouse ES cells. (A) Schematic of in vitro neural differentiation of mouse ES cells initiated by monolayer adherent culture. (B–E) Induction of neural differentiation of mouse ES cells by the monolayer adherent culture is indicated by Sox1GFP (B), Nestin (C), Tuj1 (D), and GFAP (E) positive cells. Scale bar = 200 µm. (F–H) Expressions of Sox1 (F), Nurr1 (G), and S100beta (H) in the neural differentiation of mouse ES cells were determined by quantitative real-time RT-PCR. (I) and (J) Expression of TPC2 in the neural differentiation of D3 ES cells was determined by quantitative real-time RT-PCR (I) and western blot analyses (J). The * symbols indicate the results of *t* Test analysis, *p*<0.05.

To determine whether the NAADP/TPC2 signaling is involved in the neural differentiation of ES cells, we next examined the expression pattern of TPC2 during the process of neural differentiation of ES cells initiated by monolayer culture [2] (Figure 2A). After 15-days of monolayer adherent culture treatment, 60%–70% of ES cells differentiated into neural lineages, including Sox1GFP positive neural progenitor cells (Figure 2B), nestin-positive neural progenitor cells (Figure 2C), neuron-specific class III-tubulin (TuJ1)-positive post-mitotic neurons (Figure 2D), and glial fibrillary acidic protein (GFAP)-positive astrocytes (Figure 2E). The neurogensis of ES cells was also confirmed by the mRNA expression pattern of Sox1 (Figure 2F), Nurr1 (a neuronal marker) (Figure 2G), and S100beta (a glia marker) (Figure 2H). Interestingly, *quantitative RT-PCR* and western blot analyses showed that the expression of TPC2 was initially markedly decreased after 3 days of neural differentiation, thereafter the levels of TPC2 gradually rebounded during the late stages of neuronal genesis of ES cells (Figures 2I and 2J). Yet, the TPC2 expression level was increased significantly in heterogeneously differentiated EBs, generated by aggregating ES cells in suspension (Figure S2). Taken together, these data indicated that the TPC2 expression pattern during neural differentiation is well regulated and specific, and TPC2 signaling might play a role in neural differentiation of ES cells.

### TPC2 Knockdown in Mouse ES Cells

**Figure 3:**
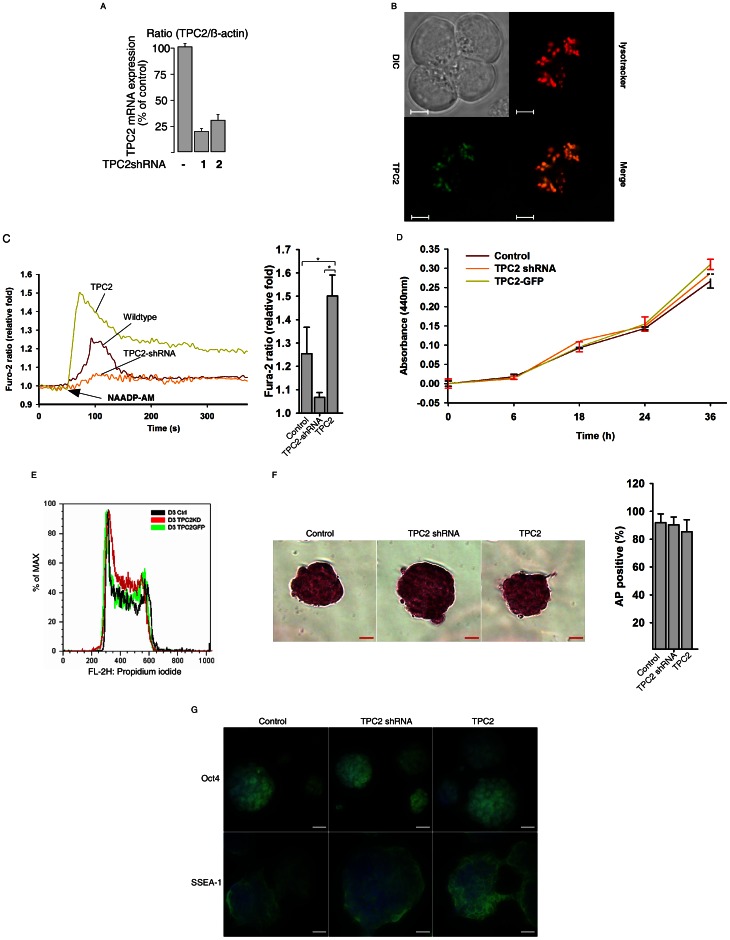
TPC2 overexpression or knockdown did not affect the self-renewal and pluripotency of mouse ES cells. (A) TPC2 knockdown by two distinctive shRNAs in D3 ES cells was verified by qRT-PCR analysis. (B) Bright field, lysotracker red staining, and GFP cell imaging of the D3 TPC2GFP cell line (Scale bar = 50 µm). (C) NAADP-AM (20 nM) induced a Ca^2+^ increase in mouse ES cells that was blocked by TPC2 knockdown but enhanced by TPC2 overexpression. Data quantifications of [Ca^2+^]_i_ peak induced by drug treatment are expressed as mean ± S.E., n = 30–40 cells. (D) WST assay of control, TPC2 knockdown, and TPC2 overexpressing D3 ES cells. (E) FACS analyses of DNA contents (PI staining) in control, TPC2 knockdown, and TPC2 overexpressing D3 ES cells. (F) Alkaline phosphatase (AP) staining of control, TPC2 knockdown, and TPC2 overexpressing D3 ES cells. Quantification of AP staining were presented as AP positive colonies/total colonies ± S.E., n = 5 (40–50 cells per experiment). (G) Immunostaining analyses of pluripotent markers, Oct4 and SSEA-1, in control, TPC2 knockdown, and TPC2 overexpressing D3 ES cells (Oct4 and SSEA-1, Green; Dapi, Blue). Scale bar = 200 µm.

We then knocked-down the expression of TPC2 in mouse ES cell lines, including D3 and 46C Sox1-GFP, by infecting the cells with lentiviruses carrying expression cassettes that encode short hairpin RNAs (shRNA) to generate gene-specific siRNAs against mouse TPC2. TPC2 mRNA or proteins were efficiently knocked-down by two different TPC2 shRNA constructs in ES cells (Figures 3A and S3). TPC2 knockdown abolished NAADP-induced cytosolic Ca^2+^ increase in ES cells (Figure 3C). Compared with the control scramble-shRNA infected ES cells, TPC2 knock-down cells exhibited no growth defects (Figures 3D and 3E), or differences in expression of alkaline phosphatase (Figure 3F) and Oct3/4 (Figures 3G and S4), which are the common markers for undifferentiated ES cells, indicating that the TPC2 knockdown cells are undifferentiated when maintained in regular ES medium.

### TPC2-GFP Expressed D3 ES Cells

Likewise, a stable TPC2-GFP (mouse) or TPC2 (rat) expressed ES cell line was established by infecting cells with lentiviruses carrying expression cassettes that encode a mouse or rat TPC2 gene (Figure S5). As shown in Figure 3B, TPC2-GFP was indeed expressed in the lysosomes as indicated by its co-localization with the lysotracker red in a subset of cellular puncta. As expected, TPC2 overexpression markedly increased NAADP-induced cytosolic Ca^2+^ increase (Figure 3C). TPC2-GFP overexpressing cells also exhibited no growth defects (Figures 3D and 3E), or differences in expression of alkaline phosphatase (Figure 3F) and Oct3/4 (Figures 3G and S4). Similar results were observed in the stable rat-TPC2 overexpressing ES cells (data not shown). Taken together, these data indicated that TPC2 overexpression also does not affect the stemness of the ES cells.

### NAADP/TPC2 Signaling Pathway Antagonized ES Cell Differentiation into Neural Progenitors

**Figure 4:**
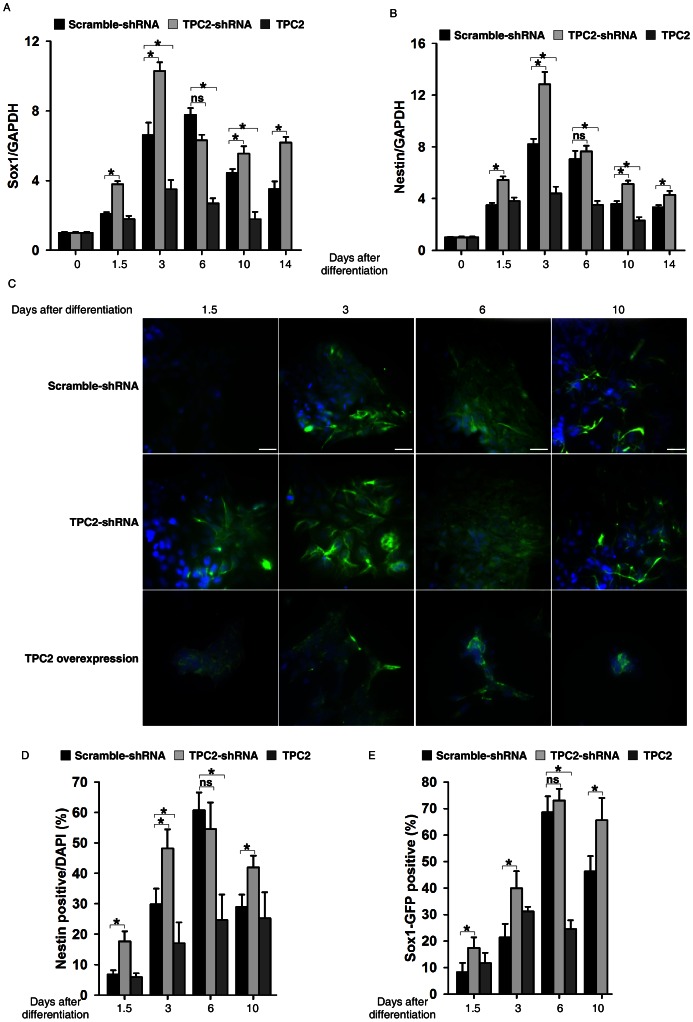
TPC2 knockdown facilitated neural lineage entry of mouse ES cells while overexpression of TPC2 inhibited it. (A) and (B) RNA was harvested at the indicated time points during neural differentiation of scramble shRNA infected, TPC2 knockdown, and TPC2 overexpressing D3 ES cells and analyzed for expression of Sox1 (A) and Nestin (B) by quantitative real-time RT-PCR. (C) Immunostaining analyses (Nestin, Green; DAPI, blue) of scramble shRNA infected, TPC2 knockdown, and TPC2 overexpressing D3 cells harvested at the indicated time point during differentiation. Scale bar = 200 µm. (D) Quantification of Nestin positive cells in (C). Data are presented as % of Nestin positive cells ± S.E., n = 5 (40–50 cells per experiment). (E) Quantification of FACS analyses of control, TPC2 knockdown, and TPC2 overexpressing 46C Sox1-GFP ES cells harvested at the indicated time point during neural differentiation. Data are expressed as mean ± S.E. from three independent experiments. The * symbols indicate the results of *t* Test analysis, *p*<0.05.

We further assessed the effects of TPC2 signaling on the early neural differentiation of mouse ES cells. TPC2 knockdown, TPC2 overexpressing, and scramble shRNA expressing ES cell lines were induced to differentiate into neural lineages following the monolayer culture protocol [2]. The expressions of Oct4 or Sox-2 during the neural differentiation of ES cells were markedly downregulated to similar levels in all three ES cell lines (Figure S6). However, quantitative RT-PCR analyses on D3 ES cells during neural differentiation showed that the expression levels of neural progenitor markers, Sox1 (Figure 4A) and Nestin (Figure 4B), were markedly up-regulated or down-regulated in TPC2 knockdown or TPC2 overexpressing cells, respectively, compared to those in control cells, especially during the early stages of differentiation. Immunostaining analyses of Nestin in wildtype, TPC2 knockdown and TPC2 overexpressing D3 ES cells derived differentiated cells (Figures 4C and 4D) supported the qRT-PCR data. Similar results were also observed by western blot analysis of Nestin (Figure S7). Furthermore, FACS analyses performed on 46C Sox1-GFP ES cells, in which GFP is targeted into the sox1 locus, during neural differentiation confirmed that more Sox1-GFP positive neural progenitors appeared in TPC2 knockdown differentiated cells than those in wildtype cells during early neural differentiation, whereas only few Sox1-GFP positive neural progenitors were detected in TPC2 (rat) overexpressing cells (Figure 4E). In summary, these results indicated that TPC2 knockdown accelerates mouse ES cell differentiation into neural progenitors, whereas TPC2 overexpression significantly blocks neural lineage entry.

### TPC2 Overexpression Caused Severe Cell Loss during Neural Differentiation of Mouse ES Cells

**Figure 5:**
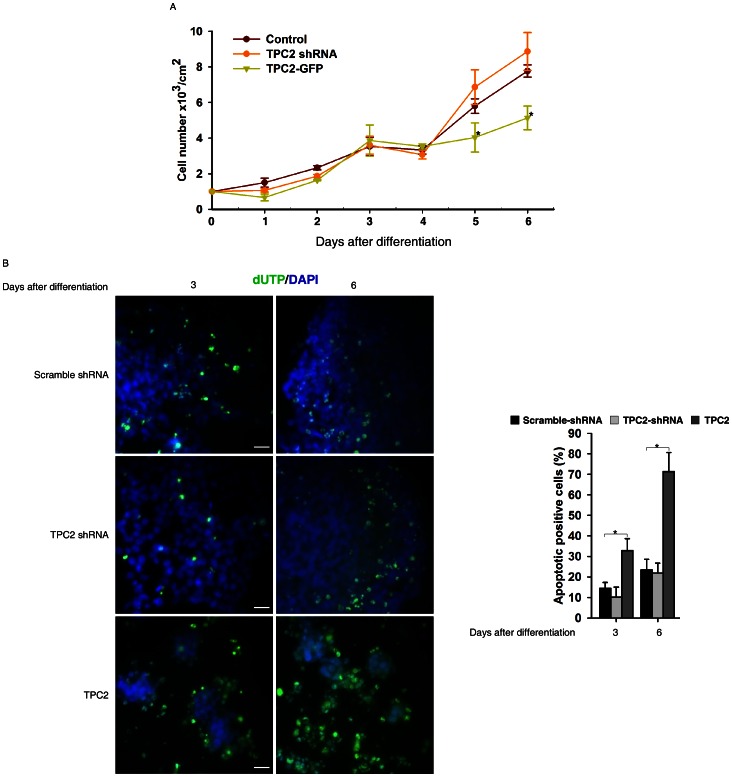
TPC2 overexpression markedly induced programmed cell death during neural differentiation of mouse ES cells. (A) Live cell number counting of control, TPC2 knockdown, and TPC2 overexpressing D3 cells at indicated time points during neural differentiation. Data are expressed as mean ± S.E. from five independent experiments. (B) TUNEL ASSAYs were performed in control, TPC2 knockdown, and TPC2 overexpressing D3 cells at indicated time point during neural differentiation (Scale bar = 200 µm). Data quantification was presented as TUNEL positive cells/DAPI-stained cells ± S.E., n = 5 (40–50 cells per experiment). The * symbols indicate the results of *t* Test analysis, *p*<0.05.

During neural differentiation of mouse ES cells initiated by monolayer culture, differentiated cells undergo dramatic cell death about 4–5 days after differentiation and majority of the survived cells adopt neural fate [2]. Interestingly, compared to control or TPC2 knockdown cells, few of TPC2 overexpressing cells survived (Figure 5A). TUNEL assay further showed that many more TPC2 overexpressing cells underwent programmed cell death during differentiation (Figures 5B and 5C). Also notably, hardly any TPC2 overexpressing cells survived to become terminally differentiated neurons or astrocytes (data not shown).

### Inhibition of Late Neuronal Differentiation of ES Cells by TPC2 Knockdown

**Figure 6:**
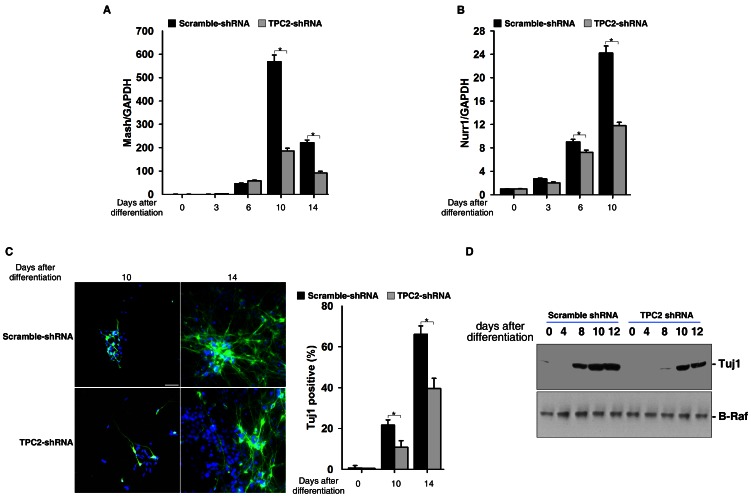
TPC2 knockdown inhibited neuronal differentiation from mouse ES cells derived neural progenitors. (A) and (B) RNA was harvested at indicated time points during neural differentiation in control and TPC2 knockdown ES cells and analyzed for expression of Mash1 (A) and Nurr1 (B) by qRT-PCR. (C) Immunofluorescent analysis of Tuj1 expression (TujI, Green; DAPI, blue) in control and TPC2 knockdown ES cells harvested at indicated time points during differentiation (Scale bar = 200 µm). Data quantification was presented as Tuj1-positive cells/DAPI-stained cells ± S.E., n = 5 (40–50 cells per experiment). The * symbols indicate the results of *t* Test analysis, *p*<0.05. (D) Cell lysates were harvested at indicated time points during neural differentiation in both control and TPC2 knockdown cells, and analyzed for expression of Tuj1 by western blot analyses.

Unexpectedly, during late stage of neural differentiation (after replating cells to PLL-laminin coated plates), there were still more Nestin- or Sox1-GFP positive cells in TPC2 knockdown cells in late (Day 10) ES-derived neural cells (Figures 4C–4E) compared to those in control cells. Paradoxically, qRT-PCR analysis showed that the expression of two neuronal makers, Mash1 (Figure 6A) and Nurr1 (Figure 6B), were down-regulated in TPC2 knockdown ES cells than those in wild type cells, especially in the later stage of differentiation. Western blot analysis on Tuj1 expression also showed that Tuj1 was down-regulated in TPC2 knockdown cells (Figure 6C). Immunostaining analyses further confirmed that fewer Tuj1 positive cells appeared in late (Day 13) ES-derived neural cells in TPC2 knockdown cells (Figure 6D). These data suggested that TPC2 deficient cells might be trapped in the neural progenitor stage and fail to progress into final neuronal stage. Since majority of TPC2 overexpressing cells died after replating and hardly any TPC2 overexpressing cells survived to become mature neurons (Figure 5 and data not shown), we were not able to assess the ability of TPC2 overexpression on late stage of neuronal differentiation of ES cells.

**Figure 7:**
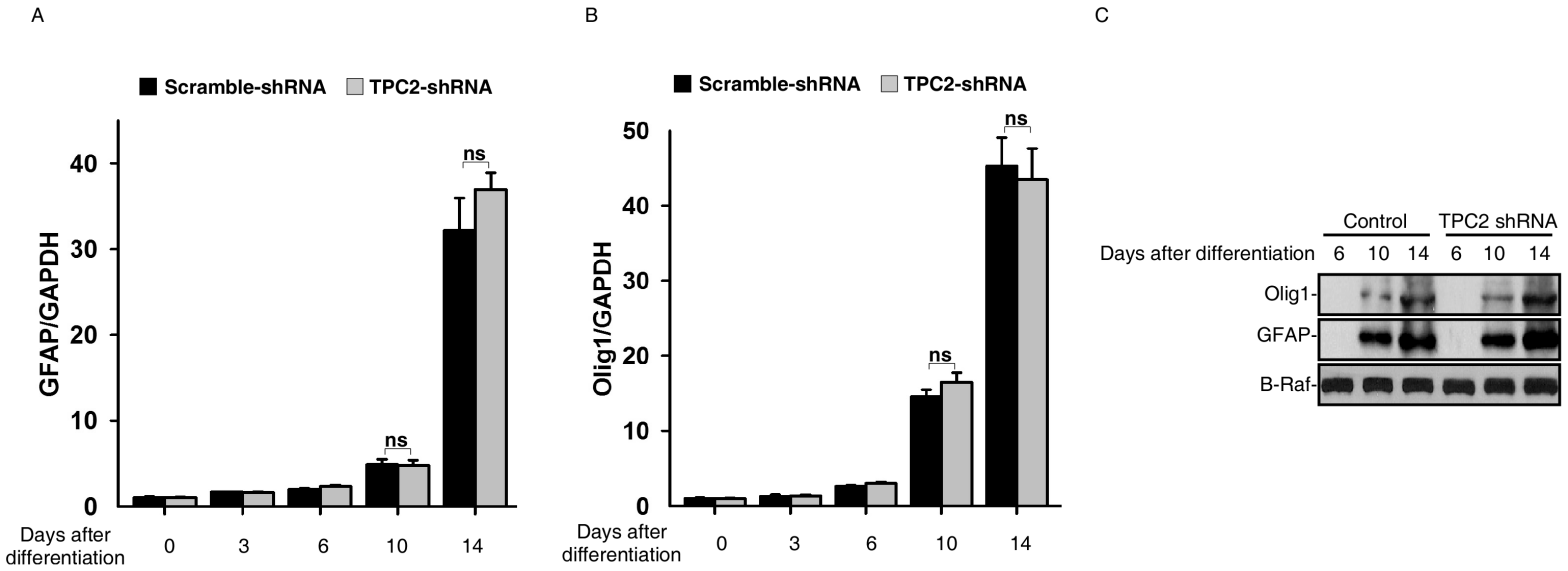
TPC2 knockdown did not affect glia differentiation of mouse ES cells. (A) and (B) RNA was harvested at indicated time points during neural differentiation and analyzed for expression of GFAP (A) and Olig2 (B) in both control and TPC2 knockdown cells by qRT-PCR. (C) Cell lysates were harvested at indicated time points during neural differentiation in both control and TPC2 knockdown cells, and analyzed for expression of GFAP and olig1 by western blot analyses.

Surprisingly, the expression of the glia makers, including GFAP and Olig2, showed no differences in wild type and TPC2 knockdown ES cells by qRT-PCR and western blot analyses (Figures 7A–7C). These data indicated that TPC2 does not play a role in the differentiation of mouse ES cells into astrocytes and oligodendrocytes.

## Discussion

The surface maker SSEA-1 and transcriptional regulator Oct4 are two widely used markers to evaluate self-renewal status of mouse ES cells [51], [52]. The expressions of SSEA-1 and Oct4 in TPC2 overexpressing or TPC2 knockdown cells were similar to those in control ES cells. In addition, the activity of alkaline phosphatase in ES cells was not affected by TPC2 knockdown or overexpression. Manipulation of the TPC2 signaling also had little effect on cell proliferation rates and cell cycle profiles of mouse ES cells (Figure 3). Taken together, these results indicate that the stemness and self-renewal of mouse ES cells are not reliant on TPC2 signaling pathway.

TPC2 was markedly down-regulated during the early stage of neural differentiation but rebounded gradually in the late stage of neurogenesis (Figures 2I and 2J). Interestingly, during EB formation, in which mouse ES cells were differentiated into various cell types [53], TPC2 expression level in the heterogeneous cell population was significantly up-regulated (Figure S2). It was also previously shown that TPC2 expression level was decreased during myogenic differentiation of C2C12 cells and primary myoblasts [54]. The diverse expression patterns of TPC2 during cell differentiation suggest that the TPC2 signaling pathway is involved in multiple cellular differentiation processes.

Indeed, we found that TPC2 knockdown facilitated mouse ES cells to differentiate into neural progenitors, whereas TPC2 overexpression inhibited mouse ES cells differentiated into neural progenitors (Figure 4). These data indicated that TPC2 signaling inhibits ES cell entry to early neural progenitors. Interestingly, the expression of TPC1, another TPC family member, is markedly increased during neural differentiation of mouse ES cells (Figure S8A). Yet, TPC1 knockdown failed to change nestin expression during neural differentiation (Figures S8A and 8B), suggesting that TPC1 is not involved in neural differentiation of ES cells. Recently, we found that NAADP/TPC2 signaling alkalinizes lysosomal pH, likely via Ca^2+^ release from lysosomes, to suppress fusion between autophagosome and lysosome, thereby suppressing autophagy progression during neural differentiation of ES cells (manuscript in preparation). We speculate that downregulation of TPC2 during early neural differentiation of ES cells facilitates the fusion between autophagosome and lysosome, thereby enabling faster energy recycling to be utilized for target differentiation. On the other hand, TPC2 overexpression blocks the fusion to prevent energy recycling and inhibits neural differentiation.

Paradoxically, we also found that TPC2 knockdown cells were trapped in the neural progenitor stage longer than that in wildtype cells (Figures 4D–4E), and the ability of TPC2 knockdown neural progenitors to differentiate into neurons was also markedly decreased compared to that of wildtype cells (Figure 6). Thus, these results suggest that neural differentiation of mouse ES cells is temporally and differentially regulated by TPC2 signaling, in that TPC2 signaling antagonizes ES cell entry to early neural progenitors, but is required for late neuronal differentiation. Along this line, it has been previously shown that NAADP but not IP_3_-mediated Ca^2+^ increases can potently induce neuronal differentiation in PC12 cells [48], [50].

We observed severe cell death during neural differentiation of TPC2 overexpressing ES cells. A TUNEL assay further showed that many more TPC2 overexpressing cells underwent programmed cell death during neural differentiation compared to control or TPC2 knockdown cells (Figure 5), and hardly any TPC2 overexpressing cells survived to the terminally differentiated cell stages, e.g. neurons and astrocytes (data not shown). Since TPC2 overexpression suppressed autophagy progression (unpublished data) and autophagy defects could lead to apoptosis [55], [56], apoptosis induced by TPC2 overexpression might be due to autophagy suppression. In addition, it has been shown previously that non-neural cells undergo apoptosis during neural differentiation under serum free conditions [57], [58]. During EB formation, TPC2 was markedly up-regulated (Figure S2), further suggesting that the TPC2 signaling is also involved in the differentiation of non-neural cell types. Thus, it is possible that TPC2 overexpressing promotes ES cell differentiation into certain non-neural cell types that cannot survive in the neural induction serum free medium, thereby undergoing programmed cell death.

We previously found that cADPR, another Ca^2+^ mobilizing messenger, promotes PC12 proliferation while inhibits its differentiation [59]. As NAADP facilitates PC12 differentiation into neurons [48], [50], cADPR and NAADP apparently play opposite roles in regulating PC12 differentiation status. Similarly, Resende *et al*. reported that Ca^2+^ signaling mediated by ryanodine receptors promotes murine embryonal carcinoma and mesenchymal stem cell differentiation into neural progenitors [60]. Here, we demonstrated that the TPC2 signaling prevents differentiation of neural progenitors from mouse ES cells. Thus these data suggest that a dedicated second messenger-mediated Ca^2+^ signaling system differentially regulates neural differentiation.

Two recent paper found that PI(3,5)P2, an endolysosome-specific phosphoinositide (PIP), activates TPC2 for Na^+^ mobilization from lysosomes, which can also be inhibited by ATP [61], [62]. They also found that NAADP cannot activate TPC2 for Ca^2+^ release from lysosomes [61], which is contrary to our data (Figures 1B, 1C, and 3C) and the work published by others [17]–[24]. Further investigations are needed to resolve the controversy. It is worthy to point out that their conclusions are largely drawn from the patch clamp studies performed on enlarged and fused endolysosmes induced by vacuolin-1 treatment. In addition, their approach to apply an extremely high concentration of NAADP-AM to induce Ca^2+^ in TPC1/2 double-knockout tissues is of concern [61]. The potential pitfall for the use of NAADP-AM is its instability [63], which may lead to non-specificity or toxicity due to its degradation. Notably, in the process of synthesizing NAADP-AM, we found the hydrobromide salt of diisopropylethyl amine (DIEA.HBr), an organic base commonly used in organic chemistry, can induce cytosolic Ca^2+^ increases by inhibiting SERCA activity via intracellular alkalinization in all cell types tested [64]. Unfortunately, it is almost impossible to completely remove DIEA.HBr in the final NAADP-AM product. Thus vigorous controls are essential when using it, and we will only use a batch of NAADP-AM when it exhibits a bell-shaped concentration response curve for Ca^2+^ release and is blocked by TPC2 knockdown or Ned-19 treatment (Figures 1B, 1C, 3C, and data not shown).

In summary, here we demonstrated a temporal and dichotomic role of TPC2 signaling pathway during neural differentiation of mouse ES cells. At early stages of neural differentiation, the TPC2 signaling pathway prevents mouse ES cells entry into neural lineage. At a later stage, TPC2 signaling is required for ES cells-derived neural progenitors differentiation into neurons, but has little effect on the differentiation of oligodendrocytes and astrocytes.

## Materials and Methods

### Cell Culture

The mouse ES cell line D3 was a gift from Prof. Tsang Suk Ying at the Chinese University of Hong Kong [65], and the 46C cell line with a GFP reporter knocked-in the Sox1 locus was a gift from Prof. Austin Smith at the University of Cambridge [2]. The cells were maintained in Dulbecco's Modified Eagle Medium (DMEM) with 15% ES qualified fetal bovine serum (FBS), 1% non-essential amino acids, 1% penicillin/streptomycin (P/S), 2 mM L-glutamate, 20 µM beta-mercaptoethanol, and 1000 unites/ml LIF on 0.1% gelatin-coated plates with feeder layers of mitomycin-c treated mouse embryonic fibroblasts (MEFs). Prior to any experimental procedures, ES cells were separated from MEFs by gravity in the suspension medium and subsequently cultured in feeder-free ES medium containing LIF on gelatin-coated plates for two passages.

### Neural Differentiation

Feeder free ES cells were plated onto 0.1% gelatin coated plates at a density of 0.8∼1×10^4^ cells/cm^2^ in N2B27 medium (1∶1 mixture of DMEM/F12 and neurobasal medium supplemented with N2 and B27, plus 50 µg/ml bovine serum albumin V, and 20 µM beta-mercaptoethanol). The medium was changed every other day until day 6. On day 6, cells were replated onto poly-l-lysine (PLL) (20 µg/ml)/laminin (20 µg/ml) coated plates at a density of 1×10^4^ cells/cm^2^. The medium was changed every two to three days until day 14.

### TPC2 shRNA and TPC2 Lentivirus Production and Infection

Two optimal 21-mers were selected in the mouse TPC2 genes (Table S1). One 21-mer was selected in the GFP gene as a control. These sequences were then cloned into pLKO.1 vector (Addgene) for expressing shRNA. Likewise, a mouse TPC2 cDNA was first amplified from the mouse cDNA pool by PCR, and was subsequently subcloned into pENTR1A-GFP-N2 (Addgene). Through a Gataway cloning system (Invitrogen), TPC2-GFP was finally recombined into a lentiviral vector, pLove (Addgene), by LR reaction. Similarly, a rat TPC2 cDNA was subcloned into a pLenti-CMV vector (Addgene). The TPC2 shRNAs, mouse TPC2GFP, or rat TPC2 lentivirus production was performed in 293T cells. For infection, ES cells were plated in 6-well plates. On the next day, 200 µL pools of concentrated shRNAs lentivirus were added to the cells in fresh medium containing 8 µg/mL polybrene. Two days later, cells infected with shRNA lentiviruses were selected in fresh medium containing puromycin (3 µg/mL) for 5 days. The puromycin-resistant cells were pooled and the knockdown efficiency was verified by quantitative real-time RT-PCR. For ES cells infected with TPC2-GFP lentiviruses, cells were trypsinized on day five after infection, washed with PBS, and sorting selection of TPC2-GFP expressed cells was then performed.

### Intracellular Ca^2+^ Measurement

Ca^2+^ measurement was performed as described previously [66], [67]. In brief, mouse ES cells were plated in 24-well plates in regular ES medium. Before the Ca^2+^ measurement, cells were incubated for 30 minutes in a dye loading buffer of 4 µM Fura-2 AM with 0.04% F127 in Hanks’ balanced salt solution in dark at room temperature. The cells were then washed with HBSS three times and incubated at room temperature for another 10 minutes. An Olympus inverted epifluorescence microscope with a CCD camera was used to capture fluorescence images obtained by alternate excitation at 340 and 380 nm with emission set at 510 nm every 3 seconds. The images were saved and analyzed by Cell R imaging software. For the NAADP-AM blocking study, either cells were preincubated with bafilomycin A1 (100 nM) for 15 minutes, or NAADP-AM (50 nM) was preincubated with esterase (50 units/ml) for 30 minutes before the Ca^2+^ measurement.

### Immunostaining Analysis

Mouse ES cells grown on coverslips at various differentiation stages were firstly washed by phosphate buffered saline (PBS) and fixed with 4% paraformaldehyde (PFA) for 20 minutes at room temperature. After another wash with PBS, cells were permeabilized with PBS containing 0.1% Triton X-100 (PBST) for 30 minutes and blocked with 1% goat serum and 1% BSA for one hour. Subsequently, the cells were incubated with primary antibodies for 2 hours and washed with PBST three times, 10 minutes each time. Secondary antibody (Alexa Fluor® 488 Goat Anti-Mouse IgG, A11008, Life Technologies, 1∶500 dilution) were then added and incubated for 1 hour. After three washes with PBST, 10 minutes each time, the cells were incubated with 4**′**,6-diamidino-2-phenylindole (DAPI) at 1∶5000 for 2 minutes. Finally, cells were fixed by Prolong Gold Antifade Reagent (Invitrogen) on slides. Slides were observed under an Olympus inverted epifluorescence microscope with a CCD camera to detect fluorescent images.

### Western Blot Analysis

Western blot analysis was performed as described previously [68]. In brief, mouse ES Cells were lysed at various differentiation stages in ice-cold EBC lysis buffer (50 mM Tris-HCl pH 8.0, 120 mM NaCl, 0.5% Nonidet P-40, 100 µM NaF, 200 µM Na_3_VO_4_, 100 µg/ml aprotinin, 20 µg/ml leupeptin, 150 µM phenylmethylsulfonyl fluoride, 0.5% sodium deoxycholate, and 0.5% SDS). Protein concentrations of each cell lysate were quantified by Bradford protein assay (Bio-Rad). Protein samples of 30 µg per lane were subjected to electrophoresis on 8∼12% SDS-polyacrylamide gels. Proteins were then transferred to Immobilon-P blotting membrane (Millipore, Billerica, MA), blocked with 5% milk in Tris-buffered saline (TBS) with 0.1% tween-20 (TBST) for 1 hour. Subsequently, the membrane was incubated with primary antibodies for 2 hours. After washing with TBST for 3 times, 10 minutes each time, the blots were then probed with specific secondary antibodies linked to HRP for 1 hour and detected by a chemoluminescence system (Millipore).

### RT-PCR and Quantitative Real-time RT-PCR

RT-PCR and quantitative real-time RT-PCR were performed as described previously [69]. Total RNA of ES cells or cells at different neural differentiation stages was extracted using RNA extraction kit (Life Technologies). RT-PCR of TPC2 using SuperScript® One-Step RT-PCR kit (Life Technologies) was performed with Takara PCR Thermal Cycler Dice (Takara). The quantitative real-time RT-PCR using the SuperScript® III Platinum® One-Step Q-RT PCR Kit (Invitrogen Life Technologies) was performed in MiniOpticon™ Real-time PCR Detection System (Bio-RAD) according to the manufacturer’s instructions. The primers used in both RT-PCR and qRT-PCR are listed in table S1.Relative gene expression was normalized to Gapdh expression.

### TUNEL Assay

Mouse ES cells grown on coverslips at various differentiation stages were collected and washed with PBS. The TUNEL assay was performed by using a TUNEL Apoptosis Detection Kit (Millipore). Briefly, cells were first fixed with 4% PFA for 30 minutes at 4°C. Cells were then incubated with biotin-dUTP and terminal deoxynucleotidyl transferase for 60 minutes at 37°C. The biotin-labeled endonucleolytic cleavage of chromatin representing apoptotic cells were detected by reaction with fluorescent conjugated avidin in dark for 30 minutes. Finally, cell nucleus was visualized by DAPI staining. Slides were observed under an Olympus inverted epifluorescence microscope with a CCD camera to capture fluorescent signals.

### WST-1 Assay

WST-1 assay (Roche) was performed on mouse ES cells grown in a 96 well plate. At each time point, WST-1 reagent of 10 µl for every 100 µl medium was added to wells and incubated for 2 hours. The reaction product, a soluble formazan salt, was detected by a microplate reader (Techan infinite M200) for absorbance at a test wavelength of 450 nm and a reference wavelength of 630 nm.

### Alkaline Phosphatase (AP) Staining

Mouse ES cells were cultured for 5 days in the mouse ES medium on coverslips under feeder free conditions. The AP staining assay was then performed by using an alkaline phosphatase detection kit (Millipore). Briefly, cells were firstly fixed with 4% PFA at room temperature for 2 minutes. Cells were then incubated with Fast Red Violet in Naphthol AS-BI phosphate solution for 15 minutes in dark at room temperature. Cells were finally washed with PBS and slides were observed under an Olympus inverted light microscope with a CCD camera to detect the signals.

### Statistical Analysis

All data were presented as mean ± standard error of mean (SEM). Student’s T-test and one-way analysis of variance (ANOVA) were performed to determine the differences among grouped data. * indicates statistically significant with P<0.05.

## Supporting Information

Figure S1 **Expressions of TPC2 mRNAs in 46C and R1 mouse ES cells were determined by RT-PCR.**
(TIF)

Figure S2 **Expressions of TPC2 mRNAs during differentiation initiated by EB formation in D3 mouse ES cells were determined by quantitative real-time RT-PCR.**
(TIF)

Figure S3 **TPC2 knockdown by shRNA in D3 ES cells was verified by western blot analysis.**
(TIF)

Figure S4 **Expressions of Oct3 and Nanog in control, TPC2 knockdown, and TPC2 overexpressing D3 ES cells were determined by western blot analysis.**
(TIF)

Figure S5 **TPC2-GFP overexpressing D3 ES cells were cultured on the MEFs feeder layers.**
(TIF)

Figure S6 **Expressions of Oct4 and Sox-2 during neural differentiation of control, TPC2 knockdown, and TPC2 overexpressing D3 ES cells were determined by western blot analyses.**
(TIF)

Figure S7 **Cell lysates were harvested at indicated time points during neural differentiation in control and TPC2 knockdown ES cells, and analyzed for expression of Nestin by western blot analysis.**
(TIF)

Figure S8 **The effects of TPC1 on neural differentiation of mouse ES cells.** (A) Expressions of TPC1 mRNAs during neural differentiation of D3 mouse ES cells were determined by qRT-PCR. (B) TPC1 knockdown by shRNA in D3 ES cells was verified by qRT-PCR analysis. (C) TPC1 knockdown had no effects on Nestin expression during neural differentiation of D3 ES cells.
(TIF)

Table S1 (DOCX)
