# Supplementary figures and images for: Two Pore Channel 2 Differentially Modulates Neural Differentiation of Mouse Embryonic Stem Cells
Source: PLoS One. 2013 Jun 12;8(6):e66077. doi: 10.1371/journal.pone.0066077 (PMC3680454; doi:10.1371/journal.pone.0066077)

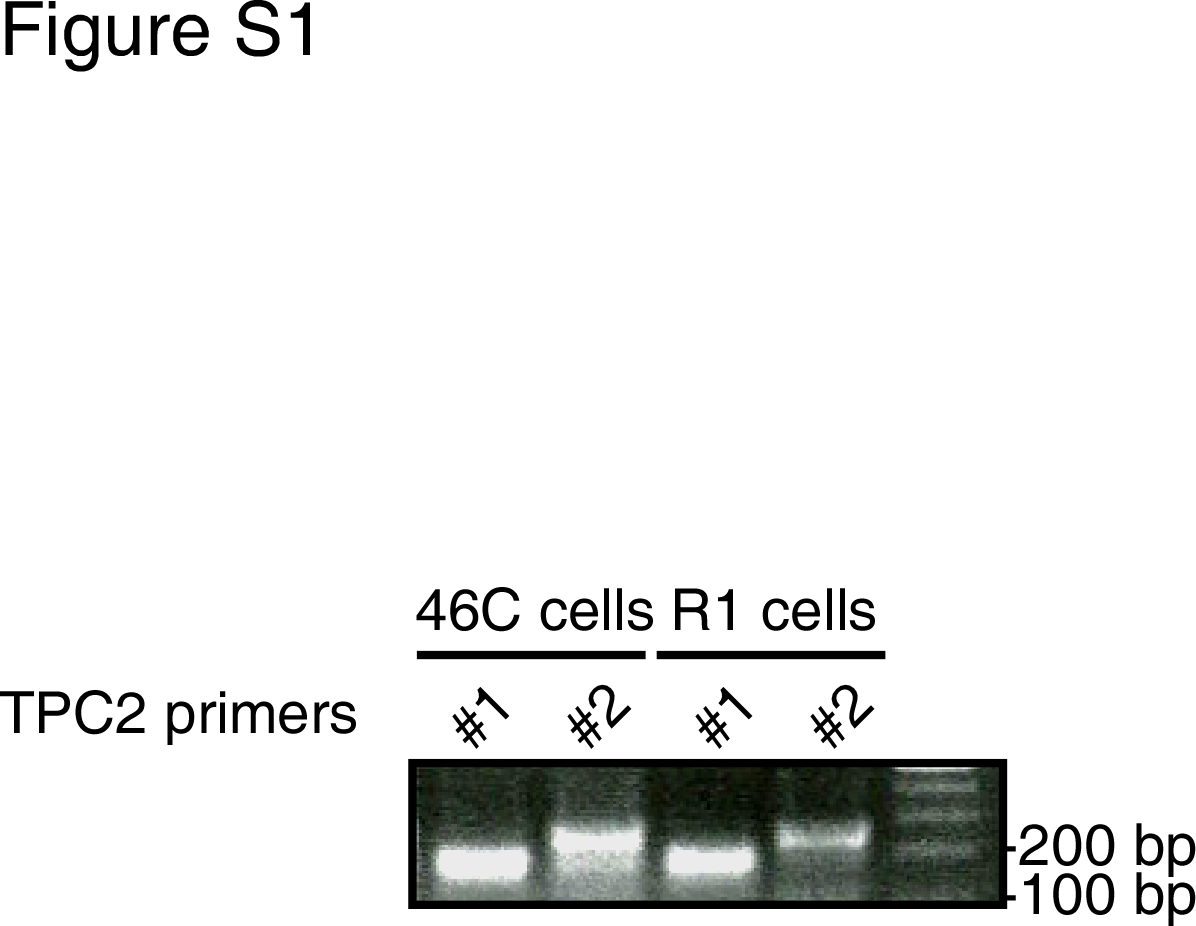

Supplement: Figure S1 — Expressions of TPC2 mRNAs in 46C and R1 mouse ES cells were determined by RT-PCR. (TIF) [file pone.0066077.s001.tif]

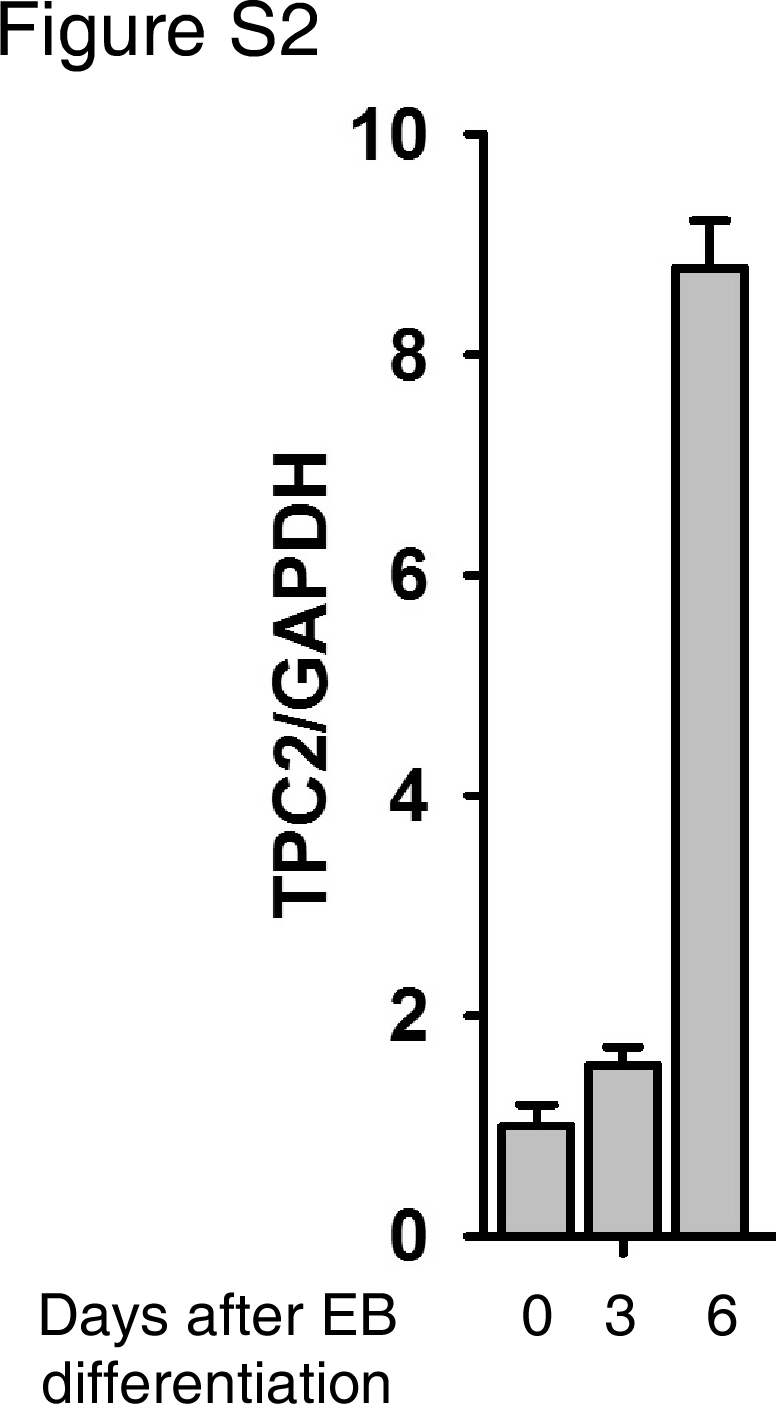

Supplement: Figure S2 — Expressions of TPC2 mRNAs during differentiation initiated by EB formation in D3 mouse ES cells were determined by quantitative real-time RT-PCR. (TIF) [file pone.0066077.s002.tif]

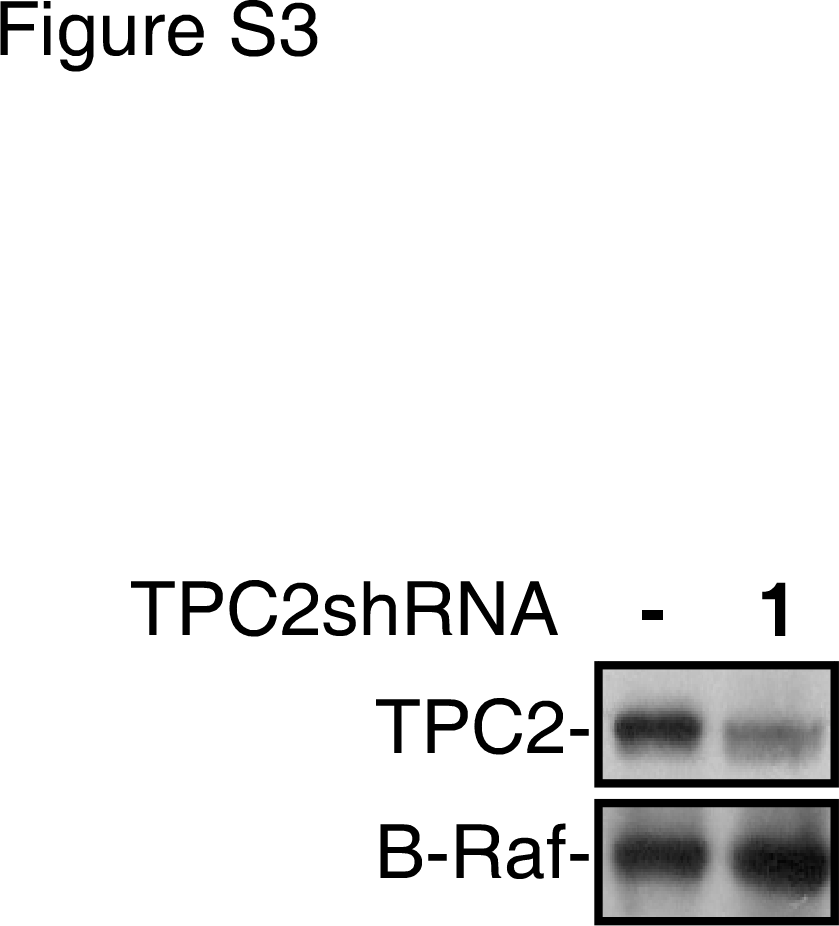

Supplement: Figure S3 — TPC2 knockdown by shRNA in D3 ES cells was verified by western blot analysis. (TIF) [file pone.0066077.s003.tif]

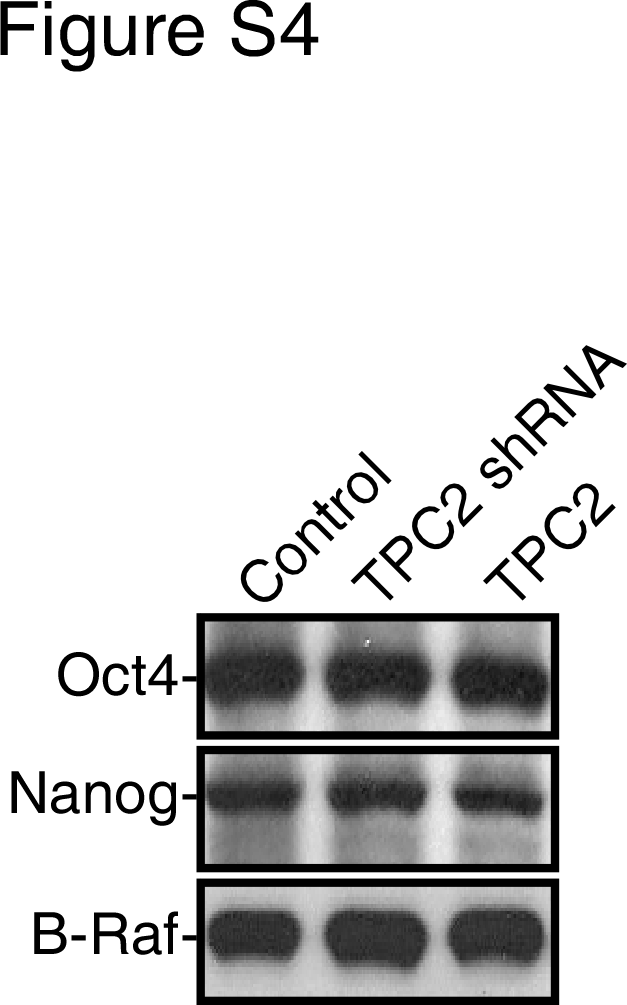

Supplement: Figure S4 — Expressions of Oct3 and Nanog in control, TPC2 knockdown, and TPC2 overexpressing D3 ES cells were determined by western blot analysis. (TIF) [file pone.0066077.s004.tif]

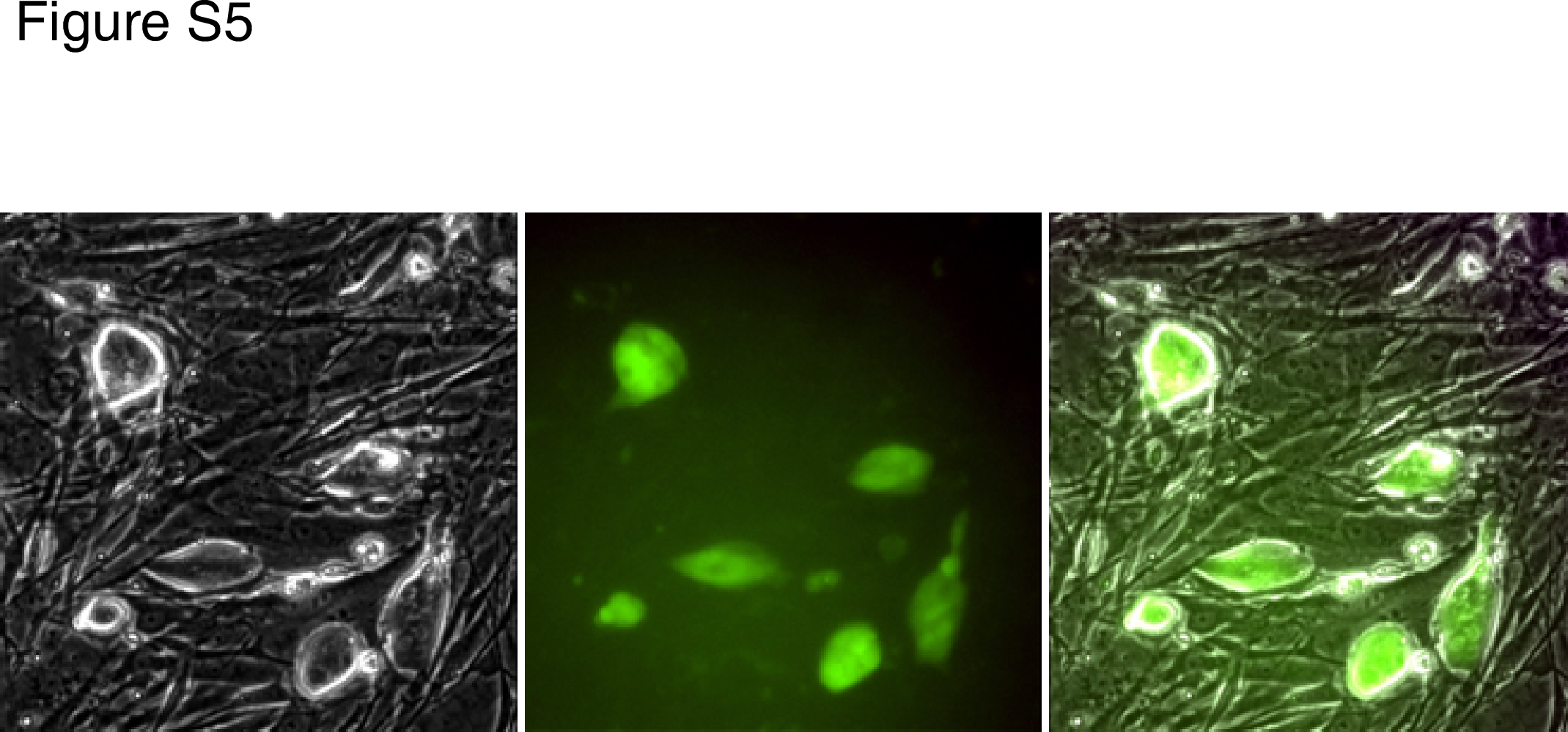

Supplement: Figure S5 — TPC2-GFP overexpressing D3 ES cells were cultured on the MEFs feeder layers. (TIF) [file pone.0066077.s005.tif]

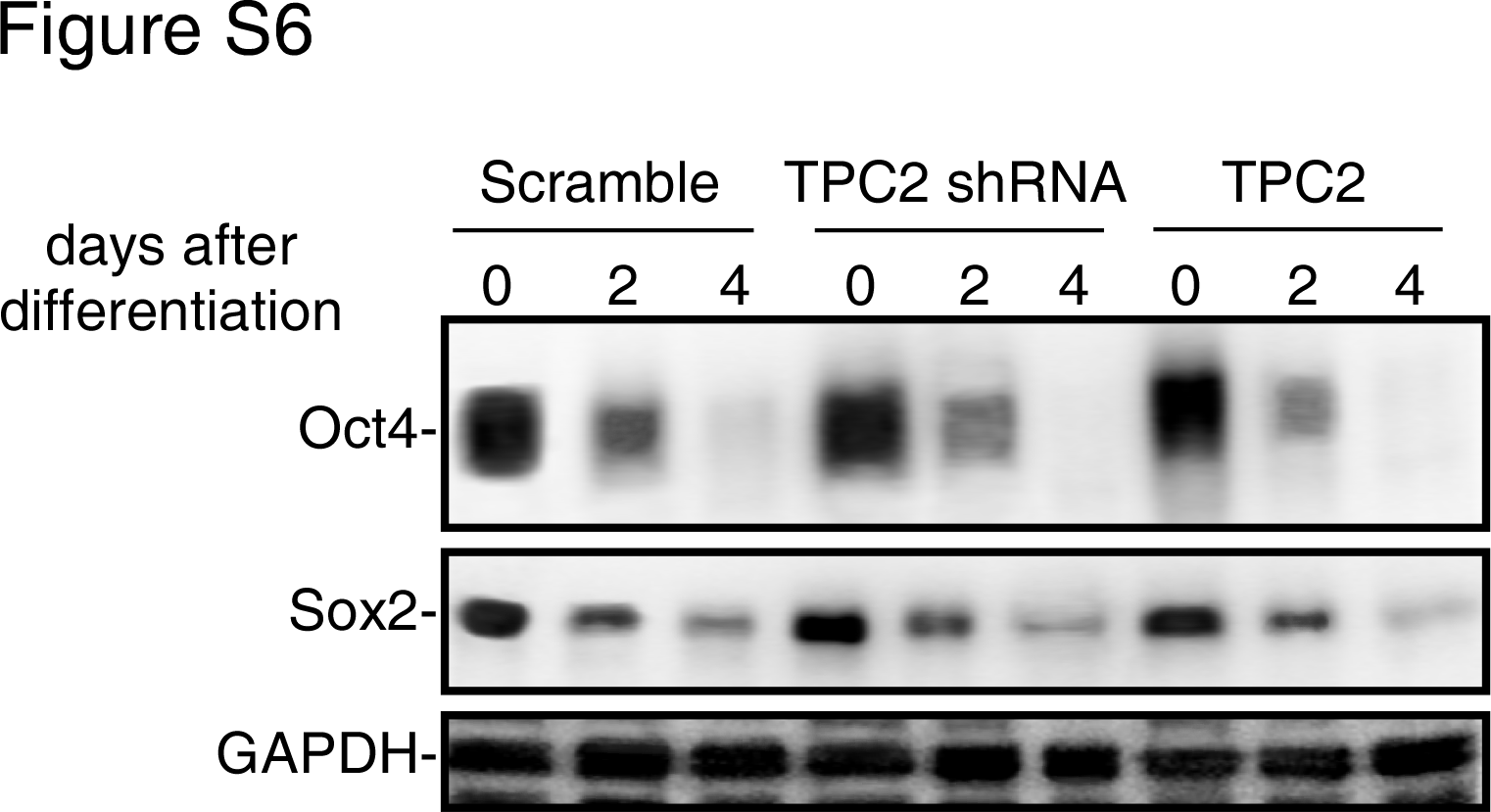

Supplement: Figure S6 — Expressions of Oct4 and Sox-2 during neural differentiation of control, TPC2 knockdown, and TPC2 overexpressing D3 ES cells were determined by western blot analyses. (TIF) [file pone.0066077.s006.tif]

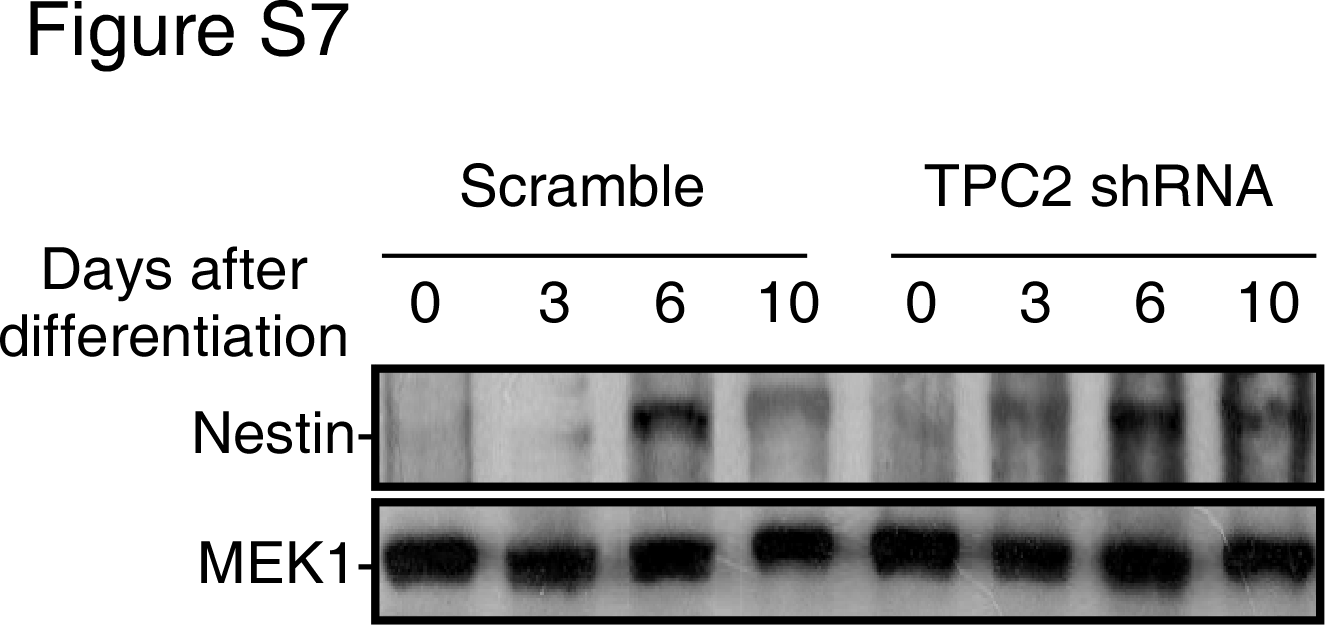

Supplement: Figure S7 — Cell lysates were harvested at indicated time points during neural differentiation in control and TPC2 knockdown ES cells, and analyzed for expression of Nestin by western blot analysis. (TIF) [file pone.0066077.s007.tif]

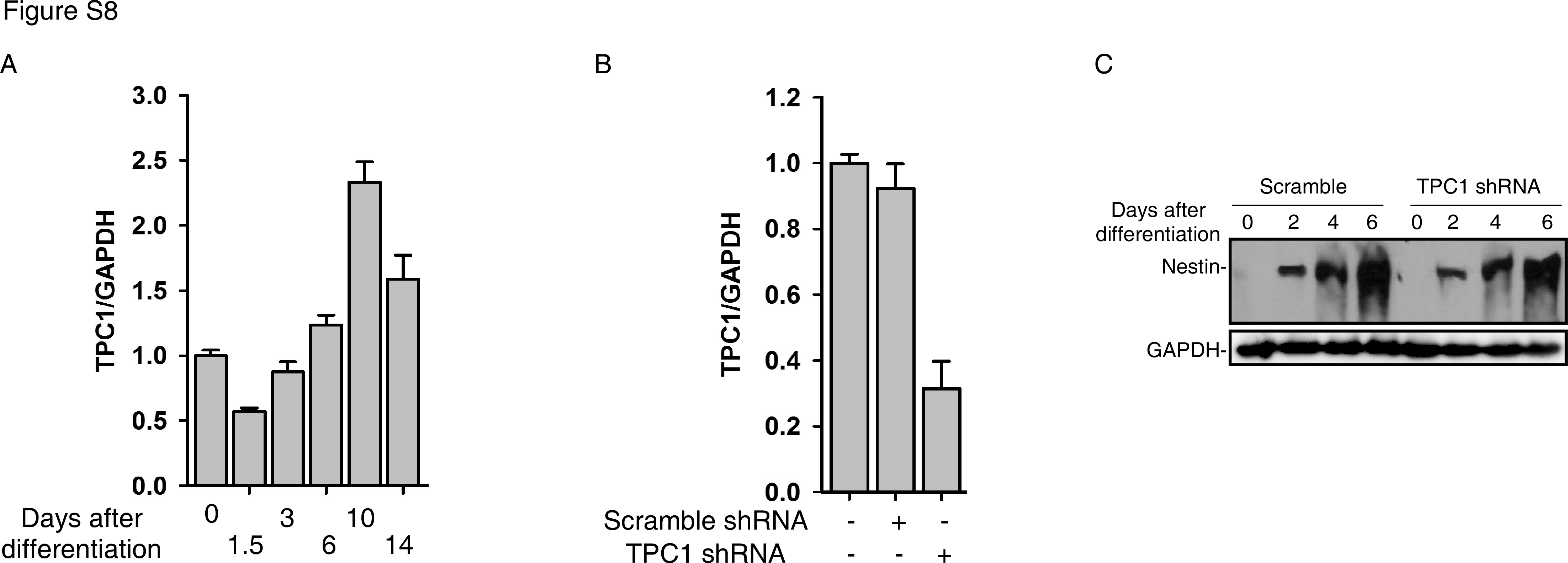

Supplement: Figure S8 — The effects of TPC1 on neural differentiation of mouse ES cells. (A) Expressions of TPC1 mRNAs during neural differentiation of D3 mouse ES cells were determined by qRT-PCR. (B) TPC1 knockdown by shRNA in D3 ES cells was verified by qRT-PCR analysis. (C) TPC1 knockdown had no effects on Nestin expression during neural differentiation of D3 ES cells. (TIF) [file pone.0066077.s008.tif]
