# Supplementary material for: Two Pore Channel 2 Differentially Modulates Neural Differentiation of Mouse Embryonic Stem Cells
Source: PLoS One. 2013 Jun 12;8(6):e66077. doi: 10.1371/journal.pone.0066077 (PMC3680454; doi:10.1371/journal.pone.0066077)
Supplement: Table S1 — (DOCX) [file pone.0066077.s009.docx]

Table S1

1. GAPDH: Forward, 5’ CTAGAGAGCTGACAGTGGGTAT;

Reverse, 5’AGACGACCAATGCGTCCAAA

1. Sox1: Forward, 5’ AGGAACACCCGGATTACAAGT;

Reverse, 5’ CGCTCATGTAGCCCTGAGAG

1. Nestin: Forward, 5’ CCCTGAAGTCGAGGAGCTG;

Reverse, 5’ CTGCTGCACCTCTAAGCGA

1. TPC2 (pair 1), Forward, 5’ CTGGTTTCGGAGATTTGCA;

Reverse, 5’ CGCAGGGATCATCACATCA

1. TPC2 (pair 2), Forward, 5’ ATACGGTGGTCGCTACCAGA;

Reverse, 5’CGCAGGGATCATCACATCA

1. Nurr1, Forward, 5’ GTGTTCAGGCGCAGTATGG;

Reverse, 5’TGGCAGTAATTTCAGTGTTGGT

1. Mash1, Forward, 5’GCAACCGGGTCAAGTTGGT;

Reverse, 5’ GTCGTTGGAGTAGTTGGGGG

1. S100beta, Forward,5’ TGGTTGCCCTCATTGATGTCT;

Reverse, 5’ CCCATCCCCATCTTCGTCC

1. GFAP, Forward,5’ GGGGCAAAAGCACCAAAGAAG;

Reverse, 5’ GGGACAACTTGTATTGTGAGCC

1. Olig2, Forward,5’ TCCCCAGAACCCGATGATCTT;

Reverse, 5’ CGTGGACGAGGACACAGTC

1. TPC2 shRNA1,

Forward, 5’ CCGGCCCAACAACTTTGACGACTTTCTCGAGAAAGTCGTCAAAGTTGTTGGGTTTTTG;

Reverse, 5’ AATTCAAAAACCCAACAACTTTGACGACTTTCTCGAGAAAGTCGTCAAAGTTGTTGGG

1. TPC2 shRNA2,

Forward, 5’ CCGGAACCTCTTGTCTATTTGTGTGCTCGAGCACACAAATAGACAAGAGGTTTTTTTG;

Reverse, 5’ AATTCAAAAAAACCTCTTGTCTATTTGTGTGCTCGAGCACACAAATAGACAAGAGGTT

1. Scramble shRNA, Forward, 5’ CCGGCCTAAGGTTAAGTCGCCCTCGCTCGAGCGAGGGCGACTTAACCTTAGGTTTTTG;

Reverse, 5’ AATTCAAAAACCTAAGGTTAAGTCGCCCTCGCTCGAGCGAGGGCGACTTAACCTTAGG
